# Stigma and the Social Burden of Neglected Tropical Diseases

**DOI:** 10.1371/journal.pntd.0000237

**Published:** 2008-05-14

**Authors:** Mitchell G. Weiss

**Affiliations:** Department of Public Health and Epidemiology, Swiss Tropical Institute, Basel, Switzerland; Swiss Tropical Institute, Switzerland

In a village in Uganda where onchocerciasis is endemic, a 25-year-old woman responded to questions about a photograph of a skin lesion presented with the story of a villager suffering from characteristic dermatitis. She described her community's experience as follows:

*“They are hiding their skin so that people cannot see them. I have not heard of anyone who wants others to know about it. No one will allow them to lead, and many people ignore them. They are considered dangerous. People fear contact with them. I feel sorry for them. Even me, I feared that from staying and meeting them we could get the disease … They find it hard to marry, and marriages can break because of this condition.”*

## Introduction

Over the past half century, social stigma has become an increasingly important topic for health social sciences. Among neglected tropical diseases (NTDs), to which I restrict my attention in this article, leprosy has been a major focus of stigma studies from the outset. Other NTDs for which stigma is an important consideration include onchocerciasis, lymphatic filariasis, plague, Buruli ulcer, leishmaniasis, and Chagas disease. Public health interest in stigma has been especially concerned with the social burden it attaches to illness, as illustrated by the account presented above. Stigma is also an important social determinant of the effectiveness of disease control through its effect on help-seeking and treatment adherence. Furthermore, stigma influences political commitment to disease control. Although that is typically a problem because stigma may encourage neglect, for agencies committed to working on problems that matter, recognition of the serious impact of stigma may encourage them to support disease control. The recent histories of onchocerciasis and lymphatic filariasis control, noted later in this article, illustrate this point.

**Figure 1:**
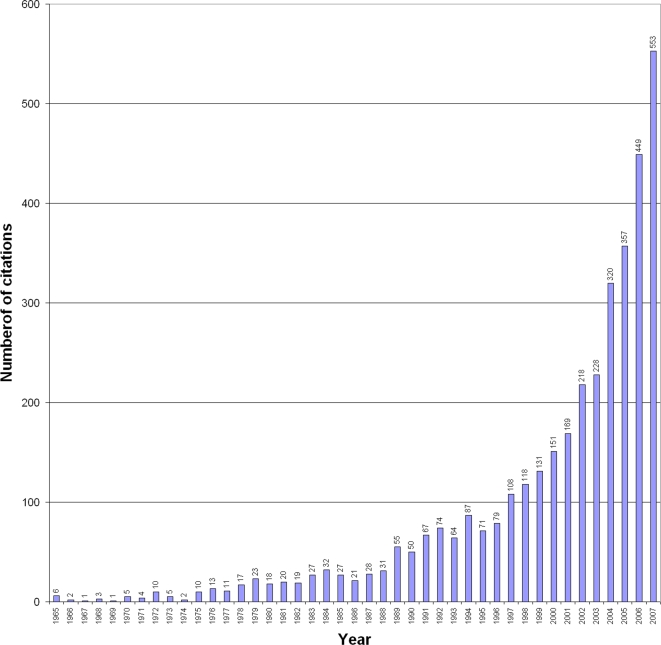
Medline Citations for Social Stigma (1965–2007). The annual number of citations for articles identified in a search for “stigma” as a text word (i.e., in the title or abstract) and excluding references to usage as a botanical term.

The impact of stigma is not readily accounted for in the epidemiological data that characterize the defined burden of disease. Instead, stigma imposes what has been termed a “hidden burden” [1]. Increasing health research interest in the topic is indicated by the literature cited in Medline. The first citation appeared in 1950, and there was no more than one citation in seven of the next 15 years to 1964. With the publication of Goffman's seminal treatise on stigma in 1963 [2], many more followed. Six citations, mostly concerned with mental health but one with leprosy stigma, are listed for 1965, and there has not been a year since then without a contribution to the health literature on social stigma. In recent years, the number has increased sharply, to 458 in 2006 (Figure 1).

Here, I address key questions about how concepts of stigma have changed over time. Who is affected, and how? What are the relevant distinctions between stigma associated with culture-specific meaning of a disease and with the social response to signs and symptoms? Current interest in the topic aims to apply answers to such questions in disease control to reduce the social burden of NTDs. Ideally, practical health social science interest aims to transform social stigma into social support. International health experience with NTDs provides some examples, and I conclude with a review of open questions for research.

## Historical Concepts of Disease-Related Stigma

Leprosy has been a major interest of health-related stigma studies from the outset. The second stigma citation in Medline documented the consistency of the harsh impact of stigma on people's lives throughout the world in Africa, Asia, the Pacific Islands, and the United States. Kellersberger in 1951 described the mistreatment of people with leprosy, highlighting misinformation that sparked fear of the disease, and tension between sensationalistic press accounts and efforts to promote responsible legislation [3]. He attributed the social stigma of leprosy to a “fear of the loathsome manifestations of the disease” and “superstitions which call down a curse from some deity.”

In presenting a social history of leprosy, Gussow and Tracy [4] questioned the scientific validity of the so-called destigmatization theory, which attributed stigma to historical, social, and medical errors, including a misreading of biblical accounts. The theory—which was promulgated by patients of the leprosy hospital in Carville, Louisiana, in their newsletter—regarded stigma as worse than the disease itself. It argued that correcting misconceptions with scientific facts about the capacity to prevent and treat leprosy would greatly reduce, if not eliminate, stigma. At that point, however, in the sulfone era when so much was still unknown about the spread and prevention of leprosy, and the capacity for effective treatment was still limited, Gussow and Tracy [4] were skeptical about whether the power of science was adequate to challenge social stigma. Better science was needed for that.

They also argued that in addition to the cultural meaning of the disease, discrediting (even racist) ideas about those who had it was also an important factor maintaining social stigma. They make that point with a rhetorical question: “What, it can be asked, might have been the status of leprosy had it been prevalent in Europe and the United States, instead of being a disease of poor people living in poor nations?” Their ensuing discussion indicates how the social history of disease and stigma foreshadowed arguments for establishing an international health focus on NTDs.

**Figure 2:**
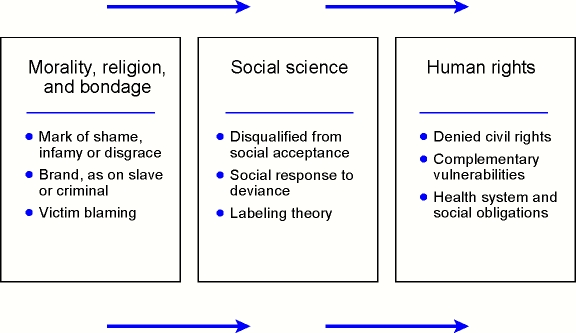
Alternative Formulations of Social Stigma.

Goffman's study of stigma reconceptualized the term with reference to social interactions, deviance, and exclusion [2]. This social formulation replaced archaic moralistic definitions that disparaged persons marked by stigma, definitions that still persist in modern dictionaries but ignore modern usage. Unlike current public health practitioners, Goffman's and other sociologists' study of stigma was primarily an academic interest. Health status was no more than a subset of a broader collection of stigmatizing conditions, and their interest was in social theory rather than social policy or health policy. Other social science theories of stigma have attempted to explain it as a product of labeling, mainly concerned with mental illness, but Nancy Waxler's analysis focused on leprosy [5]. Labeling theory, which is concerned mainly with discrimination and is relatively inattentive to other aspects of stigma [6], provided a theoretical basis for a controversial policy to rename leprosy as Hansen disease. Practical concerns that have led to rethinking health and social policy implications of stigma now emphasize the relevance of human rights as a framework for stigma studies (Figure 2). Archaic concepts that failed to question the blameworthiness or immorality of stigmatized persons have now been replaced by consideration of the immorality of unjustly denying civil rights and social acceptance of people because of their health status.

Health social science interest in stigma considers its impact not only on the experience and behavior of individuals but also on disease control [7]. The rationale that motivates stigma for different conditions includes a mix of cultural meaning, avoidance of socially discomforting disfigurement and disability, and exaggerated fear of danger and contagion. The relative influence of each of these factors varies. For leprosy and plague, the cultural meaning of the disease is an especially important feature of stigma. For other NTDs (e.g., onchocerciasis, lymphatic filariasis, Buruli ulcer, and leishmaniasis), the response to symptoms and features of the illness, and unfounded fear of contracting the condition, may operate in the absence of any clear cultural historical meaning of illness.

Measuring stigma as a guide for policy is becoming an increasing priority [8]. Although examples of stigma are often clear and striking, assessment may nevertheless be ambiguous. Some forms of discrimination are motivated by public health considerations, rather than stigma (e.g., not accepting blood donated by people with HIV). Someone excluded from a job because they have a condition, even though that condition does not prevent them from fulfilling the requirements of the position, is in a different category with regard to social stigma from someone who is excluded (removed or not hired) because they cannot fulfill the requirements of the position. On the other hand, different rules for compensation for disability from some diseases compared with others may represent a manifestation of social stigma.

Identifying factors that maintain or challenge stigma should guide efforts to mitigate its effect. Gussow and Tracy [4] argued that to end or lessen the stigma of leprosy, it is essential to understand its social history and current cultural meaning: “One cannot hope to understand the adaptational problems of patients without an understanding of the ‘world-view’ of the people involved and their view of such concepts as health and illness” [4]. Stigma affects not only patients, but also families, groups, communities and even nations, as illustrated by the “nationwide panic and a near international isolation of India” that followed the 1994 disease outbreak alleged to be plague in Surat [9],[10].

Different stigmatized conditions are also associated with distinctive features of stigma. Responses to physical deformities (e.g., edematous limbs or scrotum with lymphatic filariasis), unacceptable scratching with onchodermatitis, exaggerated concerns about the dangerousness of contagion, and moral condemnation that blames people with leprosy are all features of condition-specific stigma. Such ideas about stigma appear to be related to the experience, meaning, and behavior associated with the disease among both affected persons and unaffected persons in the community who have ideas about it and who may either stigmatize or support affected persons.

## Features and Implications of a Hidden-Distress Model

Personal experience and childhood associations may perpetuate stigmatizing social norms. Recollections of stigmatizing behavior engender fear of an anticipated social response. For example, a patient in Mumbai recently diagnosed with leprosy explained why he was so upset by the term that named his condition, despite the fact that his somatic symptoms were minimal: “My uncle has leprosy. His fingers and toes are bent like this. He can't eat or drink himself. He stays in a separate hut in the village. People keep away from him.” Such recollections lead to anticipated social exclusion: “If people were to know, they might not talk to me anymore. I would have to leave if they treated me like that. I couldn't take it” [11].

**Figure 3:**
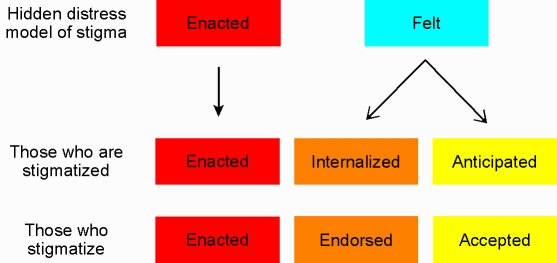
Extending Scambler's Hidden Distress Model of Stigma [12] to Facilitate Strategic Interventions.

The social rejection experienced by this patient's uncle and his fear that he might be treated similarly have been distinguished in Scambler's hidden distress model [12]. It recognizes a difference between actually experiencing discrimination or exclusion and feeling it will happen. This distinction between *enacted* and *felt* stigma may be further elaborated by differentiating *anticipated* stigma (regarded as unjustified but likely) and *internalized* stigma. In this sense, internalization refers to a process in which a person with a stigmatized condition accepts perceived exclusionary views of society and self-stigmatizes himself or herself (Figure 3).

Those who stigmatize others may do so directly or indirectly. Stigmatizers may actively engage in the process of exclusion, using their power to discriminate unfairly, ostracizing, or actively troubling someone whom they regard to be unacceptable. Others who do not actively engage in exclusion may *endorse* it, justifying and supporting exclusion though they themselves refrain, owing to legal or moral constraints. Still others may disagree with the stigmatizing behavior of their family, friends, or colleagues, but they nevertheless do nothing to stop it. They *accept* it without endorsing it, either because they feel powerless to interrupt the process, or because they feel vulnerable to stigma if they identify themselves with the interests of others who are victimized. Equating enacted stigma (a social concept) with discrimination (which may have legal implications), Deacon and colleagues further elaborate the relationship between different kinds of stigma and discrimination [6].

**Figure 4:**
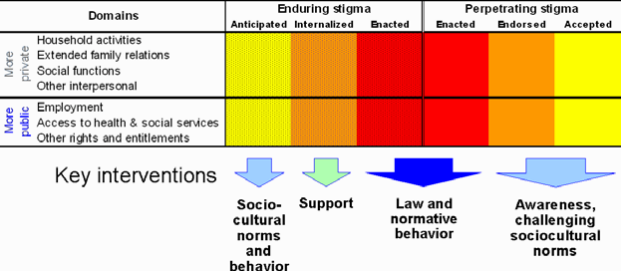
Points of Intervention to Mitigate Stigmatizing and being Stigmatized.

Manifestations of stigma, whether experienced or perpetrated, are usually situated either in a relatively more public or more private context. Public settings include schools, workplaces, and clinical health services; more private settings include social functions, family and household relations, and other interpersonal interactions. Legal protection and codes of conduct may protect people from enacted stigma in public settings or provide compensation for discrimination. Widely publicized court-awarded compensation to people with leprosy incarcerated over many years by the Government of Japan is an example [13]. Although such measures directly address enacted stigma, they are also a statement of values that may discourage endorsement and acceptance of stigmatization (Figure 4).

Complementing legal protection, promotion of public awareness of stigmatized health problems aims to challenge cultural ideas that blame victims or legitimize exclusion. It also aims to provide alternative explanations that correct exaggerated and unfounded concerns about danger and risk. Individuals who have internalized stigmatizing social views associated with their health problems benefit from support challenging these views. Such help may come from community or health care groups with a common interest and experience, from advocacy groups, or from counseling by health staff attentive to the social impact of stigmatizing illness.

## International Health Experience with Stigma and NTDs

The experience of international health projects concerned with stigma indicates how the framework presented in Figure 4 relates to local interventions. A multi-country study of onchodermatitis in Cameroon, Ghana, Nigeria, Tanzania, and Uganda used cultural epidemiological methods to examine gender-related features of the impact of stigma. Cultural epidemiology examines the distribution of categories and narrative context of illness experience, meaning, and behavior. It is particularly concerned with how such features of illness affect stigma, and effects on behavior relevant for disease control. The study of villagers with onchocercal skin disease considered their illness, and the study of unaffected residents in endemic communities used vignettes with characteristic histories and photographs depicting effects of the disease to assess the range and prominence of various community views about the condition. The respondent's account quoted at the beginning of this article was extracted from one of these studies. The approach permitted analysis of the experience and meaning of illness (patterns of distress and perceived causes) that were associated with either more or less stigma, and with gender-specific features. Findings included distinctive qualitative features of stigma for men and women. For example, men were more concerned about limitations on their economic opportunities and women with the social impact affecting prospects for marriage and family [14].

Stigma-related findings and other patterns of distress demonstrated the severity of symptoms and substantial suffering that resulted from itching, which might otherwise have been dismissed as a relatively trivial symptom. Documenting the seriousness of the condition and showing how many people were affected provided justification that helped to establish the African Programme for Onchocerciasis Control (APOC) [15]. Studies demonstrating the impact of stigma on patients with lymphatic filariasis in Ghana and Sri Lanka have also helped to document this aspect of the hidden burden of that disease [16],[17].

Addressing concerns about disability and internalized stigma arising from lymphatic filariasis, a project funded by the UNICEF/UNDP/World Bank/WHO Special Programme for Research and Training in Tropical Diseases (TDR) developed support groups in Haiti from 1998 to 2001 [18]. Disfiguring features of elephantiasis accounted for observed stigma. Young girls found it difficult to marry, and physical impairment interfered with their earning capacity. These groups integrated social support responsive to internalized stigma with practical advice and support responsive to symptoms of the disease, e.g., providing assistance for affected persons to obtain appropriate footwear. Experience showing improved self-esteem, social relations, and quality of life demonstrated the value of a group approach for integrating interventions for social stigma with other aspects of community support. The investigators suggested the approach also has broader significance for other health problems [18].

**Figure 5:**
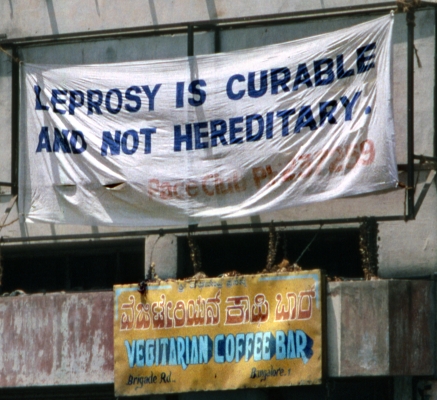
Public Health Banner to Counter Stigma through Awareness of Treatment, at Brigade Road Construction Site, Bangalore, India. Photograph: M. G. Weiss, 1992.

Program efforts in India to reduce the stigma of leprosy have also been integrated with broader interests of disease control [19]. Although we have noted that Gussow and Tracy's experience in the 1960s left them skeptical of the power of science to successfully challenge stigma at the time, subsequent developments made that question more compelling. Promoting the awareness of multidrug treatment, introduced in the early 1980s, and making it available also aimed to change ideas about leprosy that conflicted with a medical model of a treatable disease. Public awareness campaigns have focused on a simple message, that leprosy can be cured (Figure 5), delivered with greater commitment and enthusiasm than was possible when only dapsone therapy was available.

The campaign may be regarded as a response to a widely appreciated need, articulated by Gussow and Tracy, “to make leprosy ‘a disease just like any other’” [4]. They had argued that to do so, efforts to change public attitudes should be integrated with comprehensive scientific studies, “including basic scientific research, and cross-cultural medical and social epidemiological studies” [4]. Pursuing that approach in India's anti-leprosy campaign in the 1980s obligated the health system to provide effective services to ensure that the message “leprosy can be cured” was valid and credible. That experience and a continuing challenge to coordinate biomedical and social aspects of public health highlight the importance of integrating the priority of reducing disease-related stigma and other priorities for disease control.

## A Way Forward

Although hypotheses about the impact of stigma are frequently stated as proven fact, they nevertheless require testing. For example, it is frequently asserted that stigma deters help-seeking and interferes with adherence to treatment. That is true, but anecdotal examples show that stigma may also encourage treatment and promote adherence, so that a motivated patient may become free of a condition that is more undesirable because of stigma. Although that premise requires confidence that health care can help, explanatory models and illness behavior within a population also vary. We need to explain how social and cultural factors account for such a range of behavior. At another level, health policy studies need to consider how stigma influences priorities, policymaking, and health system operations.

The framework presented here suggests a research agenda appropriate for mixed-methods (qualitative and quantitative) designs of cultural epidemiology and other approaches. I hope that this framework and the experience I have reviewed may usefully guide further studies and interventions among persons with stigmatized conditions, unaffected persons in endemic communities, and among policymakers. Ultimately, stigma research encompasses an essential question that must be addressed to explain why NTDs are neglected.

**Learning Points**

1. Social stigma concerns health professionals because it contributes to suffering, may affect health-seeking and treatment adherence, and affects political commitments for disease control.
2. Archaic models of stigma—moralistic and critical of those who were stigmatized—have been supplanted by social theories based on deviance and labeling, and subsequently complemented by a formulation concerned with the priority of human rights.
3. As a guide to research and policy, the hidden distress model distinguishes *enacted* stigma from *felt* stigma. By extending that model, felt stigma may be further elaborated to distinguish *anticipated* stigma and *internalized* stigma.
4. Personal, social, health system, and policy-related interests in health-related stigma all aim to transform social stigma into social support, each in their respective domains and through means that are relevant for a particular condition and setting.
5. Variations in the experience of stigma, its effects on illness behavior, and the influence of illness explanatory models may be clarified with mixed-methods designs of cultural epidemiology and other approaches for useful studies of stigma.
